# From Adherence to Happiness: Co-designing with Frail Nursing Home Residents to Gamify Tech-based Intervention

**DOI:** 10.1093/geroni/igaf122.2802

**Published:** 2025-12-31

**Authors:** Mandi Tang, Ge Lin Kan, Mingming Fan

**Affiliations:** Hong Kong University of Science and Technology (Guangzhou), Guangzhou, Guangdong, China (People’s Republic); Hong Kong University of Science and Technology (Guangzhou), Guangzhou, Guangdong, China (People’s Republic); Hong Kong University of Science and Technology (Guangzhou), Guangzhou, Guangdong, China (People’s Republic)

## Abstract

Improving adherence to physio-cognitive interventions is crucial for mitigating frailty and dementia in older adults. Nursing home residents, often overlooked in both academic community and the marketplace, lack access to game-like experiences and report mental illness and poor adherence to interventions. To increase adherence, this article presents a three-phase co-design study with frail nursing home residents (medium age: 79 years) to gamify a physio-cognitive virtual reality (VR) intervention system, incorporating playful games in life-size VR streets and an indoor bike for self-directed, multisensory navigation. By addressing their needs for livingness, familiarity, and presence in VR-based gamification, the study demonstrates how playfulness effectively increases adherence (from 14.56 to 23.76 minutes), retention (from handful times to several weeks), and greater numbers of voluntary enrollment, while providing psychosocial benefits (p-value < 0.001). Design guidelines for VR-based interventions are proposed, with an unexpected emphasis on happiness, consistent with global efforts on dementia prevention.

